# The fish paradox: people with low socio-economic status are not consuming the right type of fish

**DOI:** 10.1093/eurpub/ckac129.169

**Published:** 2022-10-25

**Authors:** Y Zhu, JO Mierau, IJ Riphagen, LH Dekker, GJ Navis, SJL Bakker

**Affiliations:** 1 Internal Medicine, University Medical Centre Groningen, Groningen, Netherlands; 2 Medical Science, University of Groningen, Groningen, Netherlands; 3 Economics and Business, University of Groningen, Groningen, Netherlands; 4 Aletta Jacobs School of Public Health, University of Groningen, Groningen, Netherlands; 5 Certe Medical Diagnostics and Advice, Medical Center Leeuwarden, Groningen, Netherlands; 6 Public Health and the Environment, National Institute, Bilthoven, Netherlands

## Abstract

**Background:**

Fish intake is included in several national food-based dietary guidelines as a component of healthy diet because of its rich source of beneficial omega-3 fatty acids eicosapentaenoic acid (EPA) and docosahexaenoic acid (DHA). However, heterogeneity among types of fish intake is rarely studied. We investigated the associations of socio-economic status (SES) with total and types of fish intake and validated whether types of fish intake was associated with plasma EPA and DHA.

**Methods:**

From the Lifelines cohort study, 94 246 participants aged 44 ± 13 years old were included to test the association of two SES indicators, i.e., education level and income level, with dietary intakes of total, fatty, lean, fried, and other types of fish. Plasma EPA and DHA were measured in a minor subset of 575 participants (mean age: 50 ± 13 years old). Total and types of fish intake was assessed using Food Frequency Questionnaire. Linear regressions were applied, adjusted for relevant covariates.

**Results:**

After adjusting for covariates, middle and low education were negatively associated with total, fatty, lean, and other fish intake (p < 0.001 for all), and positively associated with fried fish intake (β (SE): 0.04 (0.04), p < 0.001 for middle education; 0.07 (0.04), p < 0.001 for high education), with high education as the reference group. Similar results were observed for income level. In the subset population, total and fatty fish were positively associated with plasma EPA and DHA (p < 0.02 for all). Lean and other fish intake were positively associated with only DHA (p < 0.008 for all), but not EPA, while fried fish was not associated with either EPA or DHA in plasma (p > 0.1 for all).

**Conclusions:**

Lower SES was associated with higher intake of fried fish, which did not seem to be associated with the fish-based EPA and DHA in plasma. Both nutrition education and food price policy could be implemented to increase the awareness and shape people's choice on types of fish.

**Key messages:**

• People with low socio-economic status are consuming the type of fish that is not associated with fish-based omega-3 fatty acids, so nutrition education focusing on avoidance of fried fish is needed.

• Food subsidy programs promoting intake and increasing affordability of healthier fish are needed to improve the nutritional awareness and status of our population.

